# In Vivo Regeneration of Large Bone Defects by Cross-Linked Porous Hydrogel: A Pilot Study in Mice Combining Micro Tomography, Histological Analyses, Raman Spectroscopy and Synchrotron Infrared Imaging

**DOI:** 10.3390/ma13194275

**Published:** 2020-09-25

**Authors:** Tetsuya Adachi, Francesco Boschetto, Nao Miyamoto, Toshiro Yamamoto, Elia Marin, Wenliang Zhu, Narisato Kanamura, Yoshiro Tahara, Kazunari Akiyoshi, Osam Mazda, Ichiro Nishimura, Giuseppe Pezzotti

**Affiliations:** 1 Department of Dental Medicine, Graduate School of Medical Science, Kyoto Prefectural University of Medicine, Kamigyo-ku, Kyoto 602-8566, Japan; boschetto.cesc@gmail.com (F.B.); n-miya@koto.kpu-m.ac.jp (N.M.); yamamoto@koto.kpu-m.ac.jp (T.Y.); elia-marin@kit.ac.jp (E.M.); kanamura@koto.kpu-m.ac.jp (N.K.); 2 Ceramic Physics Laboratory, Kyoto Institute of Technology, Sakyo-ku, Matsugasaki, Kyoto 606-8585, Japan; wlzhu@kit.ac.jp (W.Z.); pezzotti@kit.ac.jp (G.P.); 3 Department of Immunology, Graduate School of Medical Science, Kyoto Prefectural University of Medicine, Kamigyo-ku, 465 Kajii-cho, Kyoto 602-8566, Japan; 4 Department of Infectious Diseases, Kyoto Prefectural University of Medicine, Kamigyo-ku, 465 Kajii-cho, Kyoto 602-8566, Japan; 5 Department of Chemical Engineering and Materials Science, Doshisha University, 1-3 Tatara Miyakodani, Kyotanabe-shi, Kyoto-fu 610-0394, Japan; ytahara@mail.doshisha.ac.jp; 6 Department of Polymer Chemistry, Graduate School of Engineering, Kyoto University, Katsura, Nishikyo-ku, Kyoto 615-8510, Japan; akiyoshi@bio.polym.kyoto-u.ac.jp; 7 Division of Oral Biology and Medicine, The Jane and Jerry Weintraub Center for Reconstructive Biotechnology, UCLA School of Dentistry, Los Angeles, CA 90095, USA; inishimura@dentistry.ucla.edu; 8 Division of Advanced Prosthodontics, The Jane and Jerry Weintraub Center for Re-constructive Biotechnology, UCLA School of Dentistry, Los Angeles, CA 90095, USA

**Keywords:** spectroscopic imaging, nanogel, scaffold, bone tissue regeneration, Raman spectroscopy

## Abstract

The transplantation of engineered three-dimensional (3D) bone graft substitutes is a viable approach to the regeneration of severe bone defects. For large bone defects, an appropriate 3D scaffold may be necessary to support and stimulate bone regeneration, even when a sufficient number of cells and cell cytokines are available. In this study, we evaluated the in vivo performance of a nanogel tectonic 3D scaffold specifically developed for bone tissue engineering, referred to as nanogel cross-linked porous-freeze-dry (NanoCliP-FD) gel. Samples were characterized by a combination of micro-computed tomography scanning, Raman spectroscopy, histological analyses, and synchrotron radiation–based Fourier transform infrared spectroscopy. NanoCliP-FD gel is a modified version of a previously developed nanogel cross-linked porous (NanoCliP) gel and was designed to achieve highly improved functionality in bone mineralization. Spectroscopic imaging of the bone tissue grown in vivo upon application of NanoCliP-FD gel enables an evaluation of bone quality and can be employed to judge the feasibility of NanoCliP-FD gel scaffolding as a therapeutic modality for bone diseases associated with large bone defects.

## 1. Introduction

Elderly people frequently suffer from bone diseases associated with bone defects as a consequence of bone resorption and inactivation of the natural processes of bone remodeling [1]. Osteoporotic fracture of the spine or femoral neck may result in locomotive disability or a bedridden state. In patients receiving biological medicine treatment, osteomyelitis sometimes results in osteonecrosis, especially in the jaw, which is known as medication-related osteonecrosis of the jaw (MRONJ) [2]. Periodontal diseases may also cause tooth loss and alveolar bone defects that eventually lead to systemic infection, eating disorders, malnutrition, and dementia [3,4]. Large bone defects can also be a consequence of the surgical removal of bone tumors. All of these diseases seriously reduce patient quality of life (QOL) and activities of daily living (ADLs). It is important to counteract bone loss with the preventive preparation of sufficient bone tissue for transplantation—this remains the most critical step involved in bone regeneration surgery.

Employing tissue engineering to achieve effective bone regeneration is an alternative approach to bone grafting. This method requires a custom 3D scaffold to be built for each patient so as to precisely match the specific size and shape of the bone loss lesion. The 3D scaffold must also have an appropriate level of bioactivity so that osteoblasts can efficiently settle and produce bone matrix and minerals. Functional hydrogels are one possible biomaterial choice that were recently developed to promote bone tissue regeneration. Hydrogel scaffolds, in particular, have been demonstrated to boost cell adhesion [5,6].

“Nanogel” is the common term for a nanoscale hydrogel based on cholesterol-modified pullulan (a natural polysaccharide) [7]. Nanogel is biodegradable and completely safe to the human body. It has also been reported as a feasible drug delivery system that can transfer short interfering RNA into tumor cells easily and efficiently [8] or slowly release cytokines in vivo [9].

Nanogel cross-linked (NanoClik) gel represents an evolution of nanogel in which cholesterol is chemically cross-linked to pullulan. NanoClik gel can be formed into various sizes and shapes, and chemically functionalized for different applications [10,11,12]. To maximize its porous structure, nanogel cross-linked porous-freeze-dry (NanoCliP-FD) gel was developed by freezing and thawing NanoCliP gel [10].

In this research, we investigated the performance of NanoCliP-FD gel [13,14,15] functionalized by fibronectin and used as a scaffold carrier for KUSA-A1 mesenchymal cells. The samples were tested for three weeks in vivo on an adult murine model. The characterization was performed with an array of advanced techniques, including micro-computed tomography (μCT), Raman spectroscopic imaging, and synchrotron radiation–based Fourier transform infrared (SR-FTIR) imaging.

## 2. Materials and Methods

### 2.1. Preparation of NanoCliP-FD Gel and NanoCliP Gel

We prepared the NanoCliP gel according to a previously described method [10] using cholesterol-bearing pullulan (CHP) obtained from the NOF Corporation (Tokyo, Japan). Pullulan, which has an average molecular weight of 1 × 10^5^ g/moL, was replaced with 1.2 cholesterol moieties per 100 anhydrous glucoside units. Next, using 2-acryloyloxyethyl isocyanate (Showa Denko, Tokyo, Japan), we synthesized pullulan with acryloyl group–modified cholesterol (CHPOA). The CHPOA was then allowed to self-assemble into a nanogel. This nanogel was then cross-linked by the Michael addition of pentaerythritol tetra (mercaptoethyl) polyoxyethylene (PEGSH) with an average molecular weight of 1 × 10^4^ g/moL (NOF Corporation), resulting in the NanoClik gel [10], which was contained in microhematocrit capillary tubes (inner diameter: 0.89 mm; Thermo Fisher Scientific, Waltham, MA, USA) as a template. Highly porous NanoCliP gel was formed by freezing-induced phase separation of the NanoClik gel. NanoCliP freeze-dried matrix (NanoCliP-FD matrix) was formed from NanoCliP gel by quickly freezing it in liquid nitrogen and then drying it in a vacuum.

### 2.2. Preparation of Fibronectin-Coated NanoCliP-FD Gel

Using the procedure described in previous reports [13,14,15], a coat of fibronectin was applied to the scaffolds to stimulate cellular adhesion. This was done as follows: we obtained fibronectin solution (Wako Laboratory Chemicals, Osaka, Japan), and the NanoCliP-FD matrix was soaked in the fibronectin solution for 6 h. It was then rinsed twice in ethanol and dried. The resulting NanoCliP-FD matrix, now coated with fibronectin, was hydrated, which yielded fibronectin-coated NanoCliP-FD gel.

### 2.3. Cells

The Japanese Collection of Research Bioresources Cell Bank (JCRB, Osaka, Japan) was the source of an osteoblastic cell line, KUSA-A1 cells, which is derived from mouse bone marrow. In basic medium (Dulbecco’s minimum essential medium [DMEM]; Nacalai Tesque, Inc., Kyoto, Japan), cells were cultured. The medium was augmented with the following: 100 U/mL penicillin, 100 μg/mL streptomycin, 100 mM non-essential amino acids, and 10% fetal bovine serum (FBS). Cells were then passaged multiple times after they had formed a monolayer that was confluent. Next, the resulting KUSA-A1 cells were seeded onto NanoCliP gel at a concentration of 5.0 × 10^6^/mL and at a volume of 20 μL in a CO_2_ incubator for two hours.

### 2.4. Surgery

The US National Research Council Institute for Laboratory Animal Research’s Guide for the Care and Use of Laboratory Animals was conformed to in all our experimental procedures and protocols. All procedures and protocols in this study were approved by the Animal Research Committee or Animal Care Committee of the Kyoto Prefectural University of Medicine. Male NOG/SCID mice aged six weeks (Shimizu Laboratory Suppliers, Kyoto, Japan) were anesthetized by inhaling 1~2% isoflurane. Following this, the legs of the mice were shaved and scrubbed with a 10% solution of povidone–iodine. The skin was incised, and muscles were dissected to reveal the distal ends of the femur. The control (untreated) mice underwent the same surgical operation except for transplantation. At the diaphysis of the left femur, we created a segmental bone defect measuring approximately 1 mm × 1 mm × 6 mm using a dental drill cooled with running water. Finally, we transplanted the NanoCliP-FD gel seeded with KUSA-A1 cells into the bone defect.

### 2.5. Analyses of Micro-Computed Tomography

The mice were euthanized with a lethal dose of isoflurane 21 days after scaffold transplantation. The mice femurs were harvested and then fixed with 10% neutral-buffered formalin. The fixed samples were then observed with a TOSCANER-32300μFD micro-CT imager (Toshiba, Tokyo, Japan). The resulting data sets were reconstructed and examined using TRI/3D-BON 3D data analysis software (Ratoc System Engineering Co., Tokyo, Japan). Indices for bone structure were measured using the TRI/3D-Bon software. In order to calculate bone mineral density (BMD), we prepared a hydroxyapatite calibration curve based on images of phantoms. After calibration, we measured bone density with the CT values we had obtained. The thresholds for new bone and old bone in the mouse femur were set at 32,920 mg/cm^3^ and 33,040 mg/cm^3^, respectively.

### 2.6. Histological Analyses

Undecalcified resin-embedded femur bone specimens were deresinated with xylene for 60 min at 60 °C in order to create histological sections. The next step, Masson’s trichrome staining, was performed as follows: deresinated sections were stained with a mixed solution (10% potassium dichromate and 10% trichloroacetic acid) for 20 min, then they were treated with a solution of 1% hydrochloric acid–ethanol, stained with ponceau fuchsin for 5 min, treated with 1% acetic acid, and, finally, they were stained with a solution of phosphotungstic acid–phosphomolybdic acid. After this, the sections were stained with Aniline Blue Solution (FUJIFILM Wako Pure Chemical Corporation, Osaka, Japan) for 45 min and washed with a solution of 70 to 99.5% ethanol.

In order to perform Villanueva–Goldner staining, we treated the sections as follows: deresinated sections were stained with Cole’s hematoxylin for 10 min, treated with a solution of 1% hydrochloric acid–ethanol, stained with ponceau fuchsin for 2 min, treated with 1% acetic acid, and then stained with a solution of phosphotungstic acid–phosphomolybdic acid for 5 min. Following this, the sections were stained with Naphthol Green B Solution (FUJIFILM Wako) for 15 min and washed with a solution of 70 to 99.5%.

To perform hematoxylin and eosin (HE) staining, the deresinated sections were stained with iron hematoxylin for 20 min, treated with a solution of 1% hydrochloric acid–ethanol, stained with eosin solution, and washed with 99.5% ethanol.

After staining, the various sections were analyzed using a fluorescence microscope (BZ-X710, Keyence, Osaka, Japan) in the confocal mode.

### 2.7. Analysis of Fourier Transform Infrared Spectroscopy Based on Synchrotron Radiation (SR-FTIR)

SR-FTIR was used to evaluate the bone tissue grown on the experimental scaffolds—in particular, the content and distribution of collagen and hydroxyapatite in the bone. SR-FTIR spectromicroscopy was performed with the BL15 beam line at the Synchrotron Radiation Center at Ritsumeikan University (Kusatsu, Shiga, Japan). Resin-embedded bone tissue was sectioned into 5 μm slices. These slices were placed onto a substrate of BaF_2_ (Pier Optics Co., Ltd., Gunma, Japan). The sections were dewaxed and dehydrated, and then dried overnight in a vacuum. We used an infrared microscope (Nicolet™ Continuum™, Thermo Fisher Scientific, Waltham, MA, USA) with the following specifications: 250 μm × 250 μm liquid nitrogen–cooled MCT/A detector, 32X/NA0.65 Schwarzschild objective, motorized knife-edge aperture, and a Prior XYZ motorized stage. This was coupled with a Nicolet 6700 spectrometer (Thermo Fisher Scientific). The spectrometer had a Michelson interferometer which could record spectra with 0.4 cm^−1^ spectral resolution and 10 μm spatial resolution. We mapped the collagen and hydroxyapatite distributions with Amide I (1720–1590 cm^−1^) and PO_4_^3−^ stretching (1200–900 cm^−1^) vibrations, respectively.

### 2.8. Raman Spectroscopy

We collected Raman spectra and imaging of the bone tissue with a dedicated instrument (RAMANtouch, Nanophoton Co., Osaka, Japan). This instrument was operated in microscopic measurement mode using the 2D confocal imaging mode. Using gratings of 300 g/mm, the source of excitation was at 532 nm, the spectral resolution was 1.2 cm^−1^ (in which spectral pixel resolution equaled 0.3 cm^−1^/pixel), and an objective lens with 10× magnification with a numerical aperture of 0.3 was applied to the microprobe. We fixed the excitation laser power at 0.36 mW at the source. The Raman spectra that we acquired were deconvoluted to form Gaussian/Lorentzian curves. We performed this deconvolution with commercially available software (Origin 9.1, OriginLab Co., Northampton, MA, USA). We constructed the Raman maps with commercially available software (Raman Viewer, Nanophoton Co., Osaka, Japan).

### 2.9. Statistical Analysis

In this paper, data are expressed as means ± standard deviation. The Student’s *t*-test was used to assess significance and a *p* value of <0.05 was considered significant.

## 3. Results

### 3.1. Radiological Analysis of Bone Regeneration

**Figure 1:**
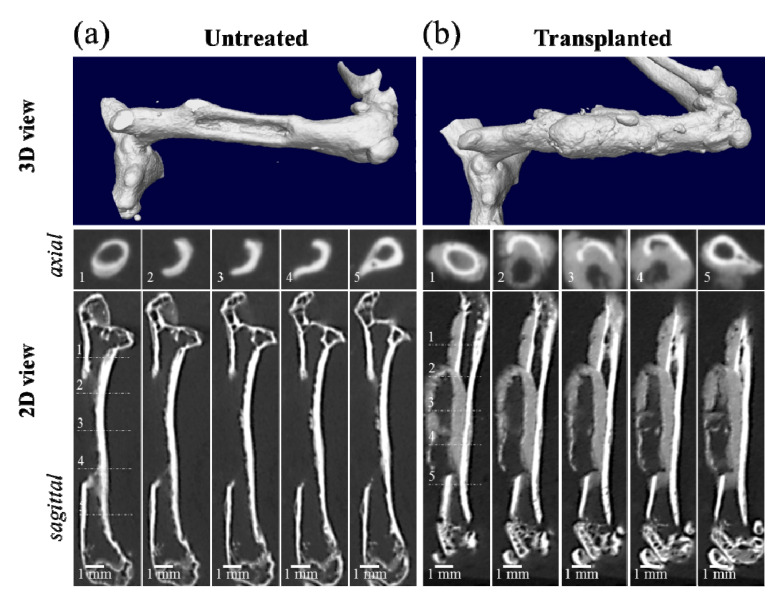
Micro-computed tomography (μCT) 3D view and a series of μCT 2D radiological images of the axial and sagittal sections of a segmental bone defect 1 mm × 1 mm × 6 mm in size at the diaphysis of the left femur of a six-week-old male NOG/SCID mouse (**a**), and a follow up 21 days after nanogel cross-linked porous-freeze-dry (NanoCliP-FD) scaffold transplantation into the bone defect (**b**). The numbers in the inset of the axial sections correspond to the respective numbers in the sagittal section.

A μCT 3D view and a series of μCT 2D radiological images of the axial and sagittal sections of the segmental bone defect at the diaphysis of the left femur of a six-week-old male NOG/SCID mouse are shown in Figure 1a. A similar imaging procedure was followed 21 days after the transplantation of a NanoCliP-FD scaffold into the bone defect (Figure 1b). A comparison between Figure 1a,b shows that new bone-like tissue formed within and around the bone defect in the NanoCliP-FD gel-transplanted sample. The radiographic features of the new bone-like tissue surrounding NanoCliP-FD suggested periosteal and endosteal reactions. The bone density was not the same as that at the cortical bone level since the regenerated bone exhibited only moderate CT contrast.

**Figure 2:**
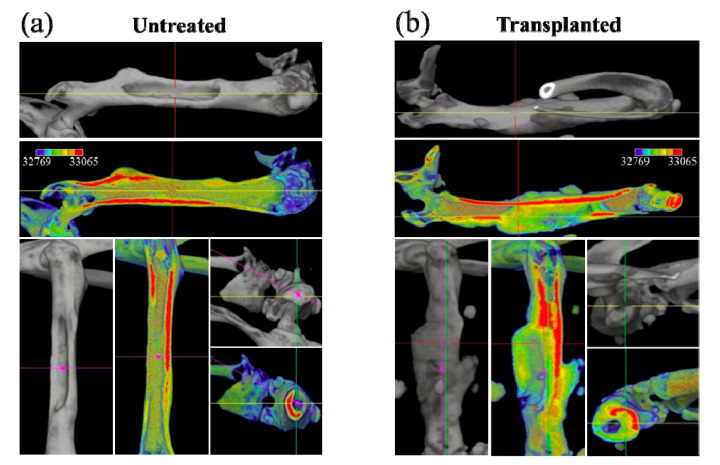
Color scale on μCT 3D views for untreated (**a**) and transplanted (**b**) femurs; red and blue colors represent the highest and lowest bone density, respectively.

As seen in the color scale of Figure 2a,b for the untreated and the transplanted bone, respectively, the μCT scans showed that, although cortical bone had the highest CT value, the regenerated bone-like tissue that surrounded the NanoCliP gel generally had a relatively lower density, with the highest values reached at the interface with cortical bone (see Figure 1b). Furthermore, due to the lack of mechanical strength of the NanoCliP-FD scaffold [13], the bone density on the remaining natural bone of the femur initially increased to compensate for the defect.

**Figure 3:**
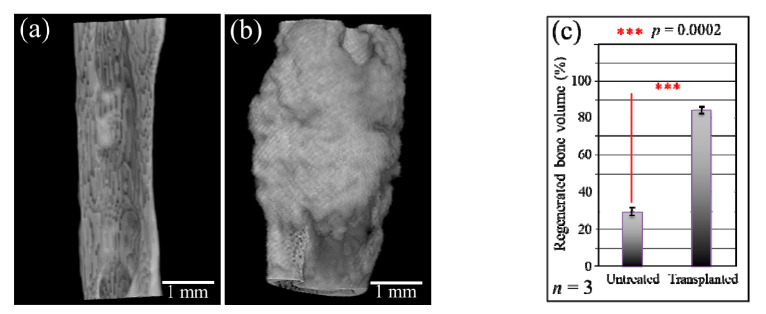
High-magnification μCT images of the bone defect before (**a**) and after (**b**) regeneration; (**c**) shows the quantitative assessment of the volume fraction of regenerated bone in the untreated and the transplanted bone.

Figure 3a,b show higher magnification μCT images of the bone defect before and after regeneration. Figure 3c shows the volume fraction of regenerated bone in the transplanted bone in comparison with natural tissue regeneration in the untreated bone (with standard deviations on a *n* = 3 sampling and statistical validation; see labels in the inset). The μCT radiological analyses in Figure 1, Figure 2 and Figure 3 clearly show the efficacy of the mesenchymal cell-treated NanoCliP-FD scaffold for the in vivo bone regeneration of large defects.

### 3.2. Histological Analyses and Raman Spectroscopic Imaging

**Figure 4:**
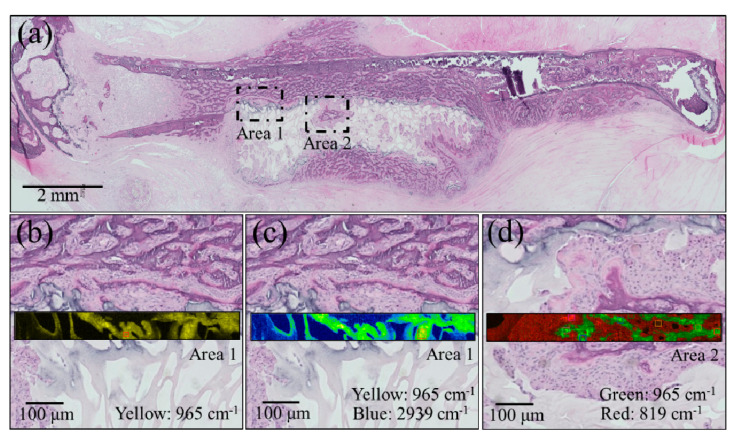
(**a**) Low-resolution histological image of the regenerated bone treated with the NanoCliP-FD scaffold; enlargements of the square insets are shown in (**b**) and (**c**) for Area 1 and in (**d**) for Area 2. In the enlarged pictures, Raman maps are shown in yellow for the intensity of the apatite mineral band PO_4_^3−^ located at ~965 cm^−1^ (**b**); for the 965 cm^−1^ PO_4_^3−^ band (yellow) and the CH_2_ region from collagen ~2939 cm^−1^ (blue); for the 965 cm^−1^ PO_4_^3−^ band (green) and the CCH band at 819 cm^−1^ (red) belonging to the collagen matrix.

After euthanizing the mice, the dissected femurs were fixed with 10% neutral-buffered formalin. Undecalcified resin-embedded histological sections were stained with HE, and bone cross sections were then analyzed with a light microscope. These histological analyses, shown in Figure 4a, suggest that the NanoCliP-FD scaffold partly retained its structure due to calcification and continued to work as a scaffold for cells while acting as a spacer that prevented the regrowth of other tissues into the bone defect. Higher resolution images taken in two different areas (see square insets in (a)) are shown in sections (b) to (d) of the same figure. These enlarged locations were also imaged by Raman spectroscopy. Raman maps that correspond to the newly grown bone at the interface with the scaffold were collected. The Raman map in Figure 4b (Area 1) shows the intensity of the apatite mineral band (i.e., the PO_4_^3−^ stretching band) located at ~965 cm^−1^ in yellow tones. The images in Figure 4c show the same zone with respect to the overlapping intensities of the 965 cm^−1^ PO_4_^3−^ stretching band (yellow) and the CH_2_ region from either collagen or the scaffold (i.e., vibrations arising from cholesterol centered at ~2939 cm^−1^ (blue)). In the Area 2 region (enlarged view in Figure 4d), the Raman map depicts the 965 cm^−1^ PO_4_^3−^ stretching band of apatite in green and the CCH in-plane bending band at 819 cm^−1^ belonging to the collagen matrix in red. The Raman maps collected according to different frequencies and in different regions of the regenerated bone consistently indicated the formation of bone mineral corresponding with the NanoCliP-FD implant, and the inside of the porosities of the scaffold, in particular, show a perfect adherence with the internal surface.

**Figure 5:**
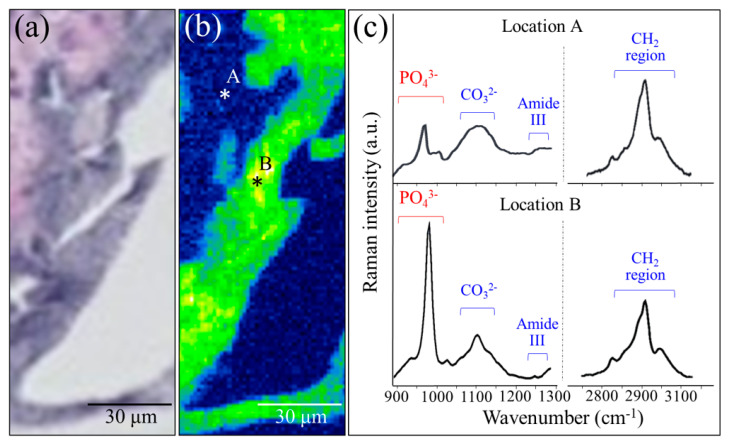
Enlarged view (**a**) and related Raman map (**b**) from a region in Figure 4c; low-and high-frequency Raman spectra collected at collagen- and apatite-rich locations (Locations A and B, respectively) are given in (**c**) (see labels in the inset).

An enlarged view and a related Raman map from the region depicted in Figure 4c are shown in Figure 5a,b, respectively. Both low-and high-frequency Raman spectra collected at collagen- and apatite-rich locations (Locations A and B, respectively) are shown in Figure 5c (see labels in the inset). These spectra additionally reveal an important detail of the bone structure, namely, the presence of the calcium carbonate (CO_3_^2−^) band at around 1100 cm^−1^. In collagen-rich locations, such as in the zones outside the NanoCliP-FD scaffold structure, such as Location A, the carbonate Raman signal was of comparable intensity to that of the apatite mineral, whereas in zones inside the scaffold, the intensity ratio between PO_4_^3−^ and CO_3_^2−^ bands was ~3.7. The latter value is lower (~25%) than that reported for femoral bone in healthy mice of similar age, meaning that the bone tissue formed on the NanoCliP-FD scaffold is rich in carbonated hydroxyapatite [16]. When compared to stoichiometric hydroxyapatite or natural bone hydroxyapatite, carbonate hydroxyapatite is associated with higher levels of osteointegration and bone remodeling [17].

**Figure 6:**
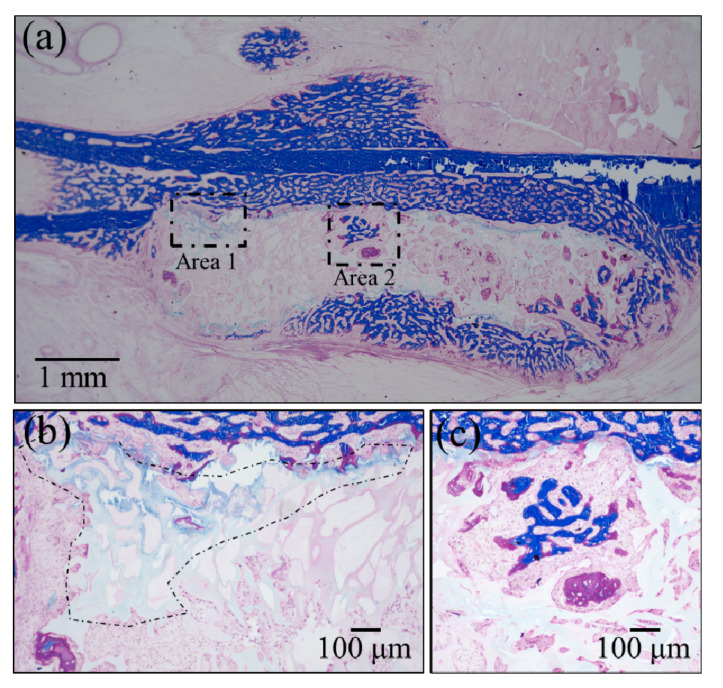
Results of Masson’s trichrome staining: (**a**) low-resolution histological image of the regenerated bone treated with the NanoCliP-FD scaffold; enlargements of the square insets are shown in (**b**) and (**c**) for Area 1 and Area 2, respectively; mature bone in deep blue, collagen in light blue, and immature bone in violet colors.

**Figure 7:**
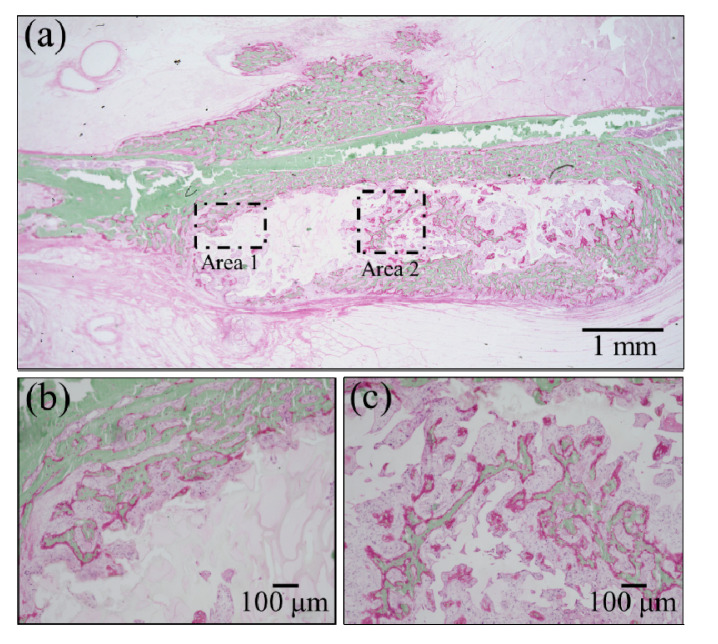
Results of Villanueva–Goldner staining: (**a**) low-resolution histological image of the regenerated bone treated with the NanoCliP-FD scaffold; enlargements of the square insets are shown in (**b**) and (**c**) for Area 1 and Area 2, respectively; mature and immature bone in green and violet, respectively.

Confirmations of the results of Raman analyses were obtained by employing two standard histological procedures, namely, Masson’s trichrome staining and Villanueva–Goldner staining, as shown in Figure 6 and Figure 7, respectively. The results of the former test displayed mature bone in deep blue, collagen in light blue, and immature bone in violet colors. A wide area was observed first (Figure 6a), and two higher-resolution images were taken in two distinct areas (i.e., the square insets in (a) labeled as Areas 1 and 2), as shown in Figure 6b,c, respectively. The results of Villanueva–Goldner staining displayed mature and immature bone in green and violet, respectively. A wide area and two enlarged locations are shown in Figure 7 (see wide area in (a) with two square insets, labeled as Areas 1 and 2 in (b) and (c), respectively). While both of the aforementioned histological procedures consistently revealed the residual presence of large areas of immature bone, it was clear that the bone tissue was undergoing a gradual shift toward a mature structure from the external part of the grafted zone toward the inner part. The “true” regenerating bone in the hydrogel showed progressive stages of bone maturation. Area 1 (within the hydrogel) might represent the early bone collagen matrix formation, and Area 2 might correspond with the last stage of hydroxyapatite formation within the bone collagen matrix. Other bone-like tissue islands in the hydrogel also appear to undergo physiological bone maturation. In sum, both enlarged areas revealed mature and immature bone areas neighboring one another, suggesting a continuous and gradual conversion from immature to mature bone tissue. These results are basically in agreement with those of the highly spatially resolved Raman spectroscopy shown in Figure 4 and Figure 5.

### 3.3. Synchrotron Radiation–Based Fourier Transform Infrared Spectroscopy Imaging

**Figure 8:**
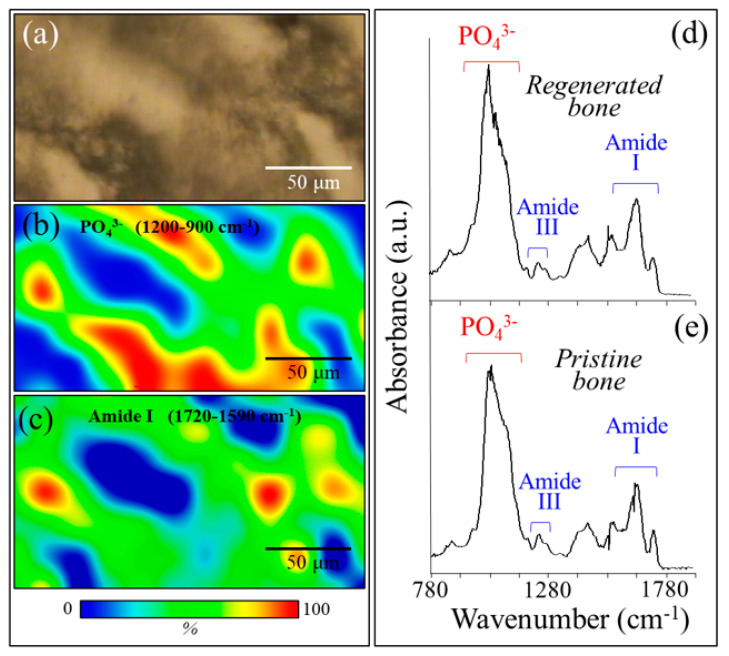
(**a**) Bright-field optical micrograph and related synchrotron radiation–based Fourier transform infrared (SR-FTIR) image of (**b**) apatite and (**c**) collagen structures in a microscopic portion within the regenerated bone scaffold area. In (**d**) and (**e**), the SR-FTIR spectra of regenerated bone are shown in correspondence with the scaffold and pristine cortical bone, respectively.

Figure 8a shows a bright-field optical micrograph, and Figure 8b,c are the related SR-FTIR images for apatite and collagen, respectively. Micrograph and SR-FTIR maps were obtained from a portion of tissue within the regenerated bone scaffold area. These results basically confirmed the morphological findings of the Raman mapping in Figure 5, showing a higher fraction of mineral apatite located in correspondence with the NanoCliP-FD scaffold structure. The mineral-to-matrix ratio, RM/M, which was calculated as the ratio between the apatite mineral band at 900 to 1200 cm^−1^ and the matrix Amide I band at 1575 to 1720 cm^−1^, was in the range of 0.60 to 1.35. This range is similar to that reported for healthy bone [18]. Figure 8d,e compare the SR-FTIR spectra of regenerated bone in correspondence with the scaffold and with pristine cortical bone, respectively. The similarity between spectra of regenerated and pristine bone tissues suggests that the NanoCliP-FD scaffold promoted the regeneration of high-quality bone.

## 4. Discussion

Several types of 3D scaffolds for bone tissue engineering have been reported in the literature [18,19], including hydroxyapatite bioceramics [20], collagen, atelocollagen, chitosan and alginate (also known as natural macromolecules and their derivatives) [21,22,23], polyethylene glycol (synthetic polymer) [24], hydrogels [25,26], and other materials, such as carbon nanotubes [26,27]. These materials have also been used in combination to achieve superior properties [19,28,29,30,31,32].

Most of those scaffolds can be formed as gel-like, sponge-like, mesh-like, sheet-like, or fibrous structures. In this context, the usefulness of NanoClik gel was already reported for tissue engineering because of its slow release of soluble factors, a property related to the nanogel character as a molecular chaperone [33,34,35]. In previous studies, we confirmed that cell adhesion could significantly be improved by imparting porosity to the NanoCliP gel by a tailored freeze dry process [10,12]. We presume that bone regeneration could become more efficient by transplanting more cells using NanoCliP-FD (porous) gel. The mechanical properties of NanoCliP gel were found improved with respect to NanoCliP-FD gel or NanoCliP-FD gel sheets [10,12]. This may be due to a high concentration of nanogel in the porous locations. Previous studies showed that soluble molecules, including BMP [32,34], FGF18 [34], EP4A [33], and W9 peptide [35], promote bone regeneration in vivo. These studies reported the unusual stimulation of periosteal progenitor cells, termed the periosteal reaction, contributing to cartilage callus [36] or fibrocartilage formation [37].

This study also suggests that NanoCliP-FD gel induced a periosteal reaction; however, it prevented periosteal tissue penetration into the graft material. The novel observation in this study is that mesenchymal stem cells coupled with the NanoCliP-FD gel scaffolds might combine the positive aspects of bone grafting and slow-release kinetics in bone regeneration within the graft material. Several independent histological and spectroscopic analyses have consistently confirmed the in vivo formation of bone with a collagen fraction similar to that of pristine healthy bone, but with a higher content of carbonated hydroxyapatite in the mineral fraction. When compared to phosphate hydroxyapatite, carbonate hydroxyapatite has demonstrated an ability to further stimulate bone remodeling [16].

The histological sections treated with Masson’s trichrome and Villanueva–Goldner staining showed substantial new bone formation within the hydrogel. The structure of the bone trabeculae of intra hydrogel bone was clearly different from that of “periosteal reaction” bone. Thus, we could safely assert that the bone tissue formed within the hydrogel was actually regenerated via mesenchymal stem cells, fibronectin, and/or hydrogel. When supplied with the necessary elements, carbonate hydroxyapatite can slowly convert back to its phosphate-rich, natural form. This suggests that NanoCliP-FD gel scaffolds provide adequate support for faster bone regeneration, which is then followed by a slow remodeling phase. A bone regeneration approach based on NanoCliP-FD gel scaffolds might offer therapeutic benefits in the reconstruction of large bone defects following surgical resection of bone tumors [38,39,40,41,42,43]. This study was limited by the lack of statistical validations of the phenomenon. We have, here, a preliminary study that shows the potential of the NanoCliP-FD gel on bone regeneration in large defects.

## 5. Conclusions

In summary, NanoCliP gel is a highly structured and bio-functionalized scaffold that enables a feasible approach to a number of clinical applications that treat diseases involving large bone loss volumes.
